# Bibliometric analysis and conversion rate of abstracts presented at the Brazilian Congress of Coloproctology into publication of full articles

**DOI:** 10.1590/0100-6991e-20233560-en

**Published:** 2023-06-22

**Authors:** HUGO SAMARTINE, DANIEL FERREIRA PAIVA, GIOVANNA BERTAZZOLA GRACITELLI, LUCAS ROSASCO MAZZINI, NICOLE GOLDENBERG LEVY, JOSE LUIS BRAGA AQUINO, ELISA DONALISIO TEIXEIRA MENDES

**Affiliations:** 1 - Pontifícia Universidade Católica de Campinas (PUC-Campinas), Faculdade de Medicina - Campinas - SP - Brasil; 2 - Pontifícia Universidade Católica de Campinas (PUC-Campinas), Programa de Pós-Graduação da PUC-Campinas - Campinas - SP - Brasil

**Keywords:** Colorectal Surgery, Bibliometrics, Meeting Abstracts, Publications, Cirurgia Colorretal, Bibliometria, Congresso, Publicações

## Abstract

**Introduction:**

the presentation of research at a congress is an interesting means for scientific dissemination, but only with publication in an indexed journal does the data become accessible and disseminated. The conversion rate in published articles of abstracts presented at congresses is an indicator to assess the scientific quality of those events. The aim of this study is to evaluate bibliometric characteristics of abstracts presented at the Brazilian Congress of Coloproctology and to determine the factors that affect publication rates.

**Methods:**

Retrospective evaluation of all abstracts presented at the Brazilian Congresses of Coloproctology from 2015 to 2019. Multiple databases were analyzed to estimate the conversion rate of the presented papers, as well as variables associated with the conversion of abstracts into full manuscripts through bivariate analysis and multivariate variables of these predictors.

**Results:**

1756 abstracts were analyzed. Most studies are retrospective, series or case reports, and even personal experience. The conversion rate was 6.9%. The presence of statistical analysis was twice as high for published abstracts as for unpublished ones.

**Conclusion:**

the data presented demonstrate a low scientific productivity of the specialty, since the research carried out is, for the most part, not published as complete manuscripts. The predictors of publication of abstracts were: multicenter studies, studies with statistical analysis, study designs with a higher level of evidence and studies awarded by the congress.

## RESUMO

**Introdução:**

a apresentação de pesquisas em um congresso é meio interessante para disseminação científica, porém apenas com a publicação em revista indexada é que os dados se tornam acessíveis e disseminados. A taxa de conversão em artigos publicados de resumos apresentados em congressos é um indicador para avaliar a qualidade científica de seus eventos. O objetivo deste estudo é avaliar características bibliométricas dos resumos apresentados no Congresso Brasileiro de Coloproctologia e determinar os fatores que afetam as taxas de publicação.

**Métodos:**

avaliação retrospectiva de todos os resumos apresentados nos Congressos Brasileiros de Coloproctologia dos anos de 2015 a 2019. Análise de múltiplas bases de dados para estimar a taxa de conversão dos trabalhos apresentados, assim como variáveis associadas à conversão dos resumos em manuscritos completos através de análises bivariadas e multivariadas desses preditores.

**Resultados:**

foram analisados 1756 resumos. A maioria dos estudos são retrospectivos, séries ou relatos de casos e até experiência pessoal. A taxa de conversão foi de 6,9%. A presença de análise estatística foi o dobro para os resumos publicados frente aos não publicados.

**Conclusão:**

os dados apresentados demonstram uma baixa produtividade científica da especialidade, já que as pesquisas realizadas não são publicadas, em sua maioria, como manuscritos completos. Os fatores preditores de publicação dos resumos foram: estudos multicêntricos, estudos contendo análise estatística, desenhos de estudo de maior nível de evidência e estudos premiados pelo congresso.

## INTRODUCTION

Paper presentations at medical conferences, whether oral or in poster format, ideally represent the vanguard of scientific knowledge, as this is where the discussion of original research themes that have not yet been published^1^. However, there is a consensus that doing research and not publishing it is similar to not having done it^2^, and research is officially disclosed and validated when it is published in indexed journals and magazines, as it is at this moment that a real methodological peer review takes place, with assessment of sample quality and originality of results. The analysis of the conversion rate of abstracts presented in full articles is one of the quality indicators for medical and scientific societies as a metric for evaluating these meetings^3^ ^-^ ^9^.

The Brazilian Society of Coloproctology (SBCP) is the second society in the area in number of members in the world, but the conversion rate from abstracts presented to full articles in its scientific events has not yet been analyzed. Every year, approximately two thousand people from all over the world attend the main congress of the specialty in the country. The annals published as supplements to the Journal of Coloproctology (JCOL) expose at least 500 abstracts of papers presented as free-themes, posters, and free-videos. The fact of knowing the characteristics of these published articles allows a critical analysis of the quality of scientific production and the proposition of possible planning changes to be conducted both by the society that represents the specialty and by the academic community and governmental actions to promote research^6^ ^,^ ^10^ ^-^ ^12^.

The aim of this study is to analyze the conversion rate of abstracts presented at the Brazilian Congress of Coloproctology and to evaluate the predictive factors for publication of such abstracts.

## METHODS

### Abstracts Collection

We conducted a descriptive analysis through a bibliometric study of the abstracts presented at the Brazilian Congress of Coloproctology from 2015 to 2019, using the annals available on the SCBP website^13^. We included oral presentations/free themes and posters, excluding presentations in free-video format (291). We also excluded abstracts that were not complete, those whose title did not match the content of the text, those that were repeated, and those that lacked authors’ information (33).

Two different examiners used a standard form for data collection, using the Microsoft Excel 2019 software. To ensure the uniformity of the analyzes, the reviewers initially evaluated 15 (fifteen) abstracts from each year as a test in a calibration meeting. Reliability intervals between different investigators were not used, as all discrepancies and/or conflicts were separated and subsequently discussed in regular meetings until a consensus was reached.

### Variables Studied

We evaluated the following information in the abstracts: year of presentation; type of presentation: oral or poster; title; awarded at the congress where it was presented; belonging to a university center; number of authors; study design; number of patients involved; uni or multi-center study; presence of statistical analysis (excluding case reports); published as a scientific article; and presentation in any other Brazilian congress of Coloproctology.

For category definition, we used the same options offered for submitting papers at the respective congresses: benign anorectal diseases; malignant and premalignant diseases of the colon, rectum, and anus; inflammatory bowel diseases; pelvic floor diseases and intestinal and anorectal physiology; experimental studies in Coloproctology; sexually transmitted diseases; colonoscopy; and miscellaneous.

### Research of Published Manuscripts

**Figure 1:**
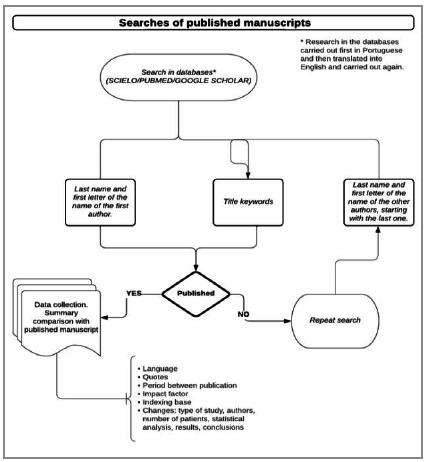
Methodology flowchart for researching full manuscripts.

We identified publications in peer-reviewed journals through a standardized search of the MEDLINE (PubMed), SciELO, and Google Scholar databases from March to November 2021. We used combinations of the last name and the first letter of the first name of the first author of abstracts associated with title keywords in Portuguese and English. If no exact match was found or if there were no results for a search, the process was repeated using the other authors of the abstracts, starting with the last one, according to Figure 1. If the result included no publications or several publications with the same author, we increased the search criteria by the title, abstract text keywords, or another author’s name. Whenever a peer-reviewed manuscript was retrieved, we compared the information contained in the abstract and the manuscript to determine if they matched according to previously applied stringent criteria^7^ ^,^ ^8^ ^,^ ^11^ ^,^ ^14^ ^,^ ^15^.

For each corresponding abstract/manuscript, we recorded the following data, as shown in Figure 1: title, period between publication and presentation of the abstract (previous, <12 months, 12-24 months, >24 months); journal name; journal indexing (Web of Sciences; MEDLINE; SciELO; LILACS, others); national or international; form of access (free, login, or paid access); impact factor of the journal on the date of publication, according to SCIMAGO - Journal & Country Rank and Thompson Reuters Journal Citation Report^®^ and H-index; publication language (English, Portuguese, or both); and the number of citations of the manuscript according to Google Scholar and/or Web of Sciences, if indexed in this database^6^ ^-^ ^8^ ^,^ ^11^ ^,^ ^14^.

We also included articles with a publication date prior to presentation at the evaluated congress. We excluded from the sample publications in the annals of various congresses, symposiums, workshops, books, or any other means of publication other than peer-reviewed journals^16^. We did not contact the authors and co-authors of the unpublished abstracts, and it was also not allowed to assess the reason why they were not successfully published^1^.

### Statistical analysis

For the descriptive analysis, we used the mean for numeric variables and percentage for categorical ones. For quantitative data in the comparison between years, we used the ANOVA test. We defined the conversion rate as the ratio between the number of manuscripts published in peer-reviewed journals and the total number of abstracts presented at conferences. We performed intra and interperiod comparisons. We applied Analysis of Variance, Equality of Two Proportions, Student’s Paired T-test, Chi-Square test, and Confidence Intervals for the Mean in the statistical comparisons. We conducted bivariate analysis and multivariate logistic regression to determine which independent abstract variables (number of authors, number of subjects, coloproctology topics, presence of statistical analysis, and study designs) were significant predictors of conversion of abstracts into full manuscripts (dependent variable). All analyzes were performed using the Statistical Package for Social Science software (SPSS version 20.0 for Windows, Chicago, IL, USA). Values were considered significant for a 95% confidence interval and p<0.05^7^ ^,^ ^15^ ^,^ ^17^.

## RESULTS

### Abstract Results

We analyzed 1,756 abstracts presented at the Brazilian Congress of Coloproctology from 2015 to 2019, with the characteristics described in Table 1. The majority, 1,169 (66.6%), were presented in the poster category and 33.4% in the oral category (Table 1). There was heterogeneity in the number of abstracts presented per year, as well as the presentation format. It is noteworthy that the area that received the most presentations of abstracts was malignant and pre-malignant diseases of the colon, rectum, and anus, with 34.3%. In addition, 59.7% belonged to a university center and only 8.4% of the studies were multicenter. On the other hand, half of the abstracts (49.4%) were case reports, with an increase in this proportion in the last two years (2018: 55.2% and 2019: 59.3%).

**Table 1:** Characteristics of abstracts presented at the Brazilian Congress of Coloproctology from 2015 to 2019 and bivariate analysis of factors related to publication.

Variables	Not published		Published		Total		p-value
	n	%	n	%	n	%	
Category							
Oral	512	31.3	75	62	587	33.4	<0.001
Poster	1.123	68.7	46	38	1.169	66.6	
Multicentric							
No	1504	92	103	85.8	1.607	91.6	0.019
Yes	131	8	17	14.2	148	8.4	
University center							
No	657	40.20%	50	41.30%	707	40.30%	0.805
Yes	978	59.80%	71	58.70%	1.049	59.70%	
Awarded							
No	890	98.9	84	93.3	974	98.4	<0.001
Yes	10	1.1	6	6.7	16	1.6	
Presence of statistical analysis							
No	538	68.3	52	50.9	590	66.3	
Yes	249	31.7	50	49.1	299	33.7	
Presence of women							
No	91	7.4	21	24.8	112	8.4	
Yes	1.140	92.6	84	75.2	1.224	91.6	
Presented at previous congresses							
No	1.592	97.40%	105	86.80%	1.697	96.60%	
Yes	43	2.60%	16	13.20%	59	3.40%	
Number of authors							
1-3	91	5.60%	13	10.70%	104	5.90%	
4-5	185	11.30%	12	9.90%	197	11.20%	
≥6	1.359	83.10%	96	79.30%	1.455	82.90%	
Area							
Colonoscopy	97	5.90%	4	3.30%	101	5.80%	0.142
Benign Anorectal Diseases	180	11.00%	17	14.00%	197	11.20%	
Pelvic floor diseases/Intestinal and Anorectocolic Physiology	130	8.00%	12	9.90%	142	8.10%	
Inflammatory Bowel Diseases	255	15.60%	15	12.40%	270	15.40%	
Malignant and premalignant diseases of the colon/ rectum and anus	558	34.10%	44	36.40%	602	34.30%	
Sexually Transmitted Diseases	32	2.00%	2	1.70%	34	1.90%	
Experimental Studies in Coloproctology	28	1.70%	6	5.00%	34	1.90%	
Miscellaneous	355	21.70%	21	17.40%	376	21.40%	
Variables	Not published		Published		Total		p-value
	n	%	n	%	n	%	
Study design							
RCT	4	0.2	0	0	4	0.2	<0.001
Experimental studies	40	2.4	11	9.1	51	2.9	
Others	23	1.4	1	0.8	24	1.4	
Prospective	188	11.5	39	32.2	227	12.9	
Case report	848	51.9	19	15.7	867	49.4	
Retrospective	482	29.5	44	36.4	526	30	
Literature review without systematic review	4	0.2	0	0	4	0.2	
Systematic review	7	0.4	1	0.8	8	0.5	
Case series	39	2.4	6	5	45	2.6	

*Analysis performed using the Chi-Square test; RCT: Randomized Clinical Trial.*

Statistical analysis was present in only 33.6% of the works; 59 abstracts (3.4%) had already been presented at the Brazilian Congress of Coloproctology in other years.

### Results of Published Works

We found 121 abstracts published as articles, which represents a conversion rate of 6.9% for full manuscripts when we analyze the five years grouped together. However, there was variation in the conversion rate (p-value <0.001) when analyzed year by year: 2015: 11.4%; 2016: 10.6%; 2017: 5.7%; 2018: 4.3%; 2019: 3.8%. Table 2 brings the characteristics of the published works.

**Table 2:** Qualitative factors of works presented and published in complete articles.

Variables	n	%	p -value
Published			
No	1,635	93.1	Ref.
Yes	121	6.9	<0.001
Time category			
Previous	39	32.2	Ref.
<12 months	34	28.1	0.484
12-24 months	34	28.1	0.484
≥25 months	14	11.6	<0.001
Evidence level			
Grade 2	41	33.9	0.503
Grade 3	46	38.0	Ref.
Grade 4	3	2.5	<0.001
Grade 5	31	25.6	0.038
Presence of statistical analysis			
No	26	25.70	<0.001
Yes	75	74.30	Ref.
Citations from the published study			
No	46	38.0	<0.001
Yes	75	62.0	Ref.
National / International			
International	43	35.5	<0.001
Ref.			
National	78	64.5	
Access			
Free access	104	86.0	Ref.
Free with login	3	2.5	<0.001
Paid	14	11.6	<0.001
Variables	n	%	p -value
Index Base Category			
1-3	8	6.6	<0.001
4-5	66	54.5	Ref.
≥6	44	36.4	0.005
Zero	3	2.5	<0.001
PubMed			
No	60	49.6	0.898
Ref.			
Yes	61	50.4	
LILACS			
No	54	44.6	0.095
Ref.			
Yes	67	55.4	
SciELO			
No	58	47.9	0.52
Ref.			
Yes	63	52.1	
Web of science			
No	97	80.2	Ref.
<0.001			
Yes	24	19.8	
Scopus			
No	22	18.2	<0.001
Ref.			
Yes	99	81.8	
Language			
Spanish	1	0.8	<0.001
English	98	81.0	Ref.
Portuguese	7	5.8	<0.001
Portuguese and English	15	12.4	<0.001
Number of authors			
1-3	17	14.0	<0.001
4-5	21	17.4	<0.001
≥6	83	68.6	Ref.
Study design			
Experimental studies	11	9.1	<0.001
Prospective	39	32.2	0.346
Case report	20	16.5	<0.001
Retrospective	46	38.0	Ref.
Literature review without systematic review	2	1.7	<0.001
Case series	3	2.5	<0.001

*Analysis performed using the equality of two proportions test. Notes: Ref.: Reference; LILACS: Latin American and Caribbean Literature in Health Sciences; SciELO: Scientific Electronic Library Online. Source: Author (2022).*

The average time for publication was 16.1±3.08 months, with emphasis on the SBCP journal itself, with 52 of the 121 articles published from the abstracts (Table 3). Journals with only one published article were not listed, which were grouped in “Others”.

**Table 3:** Distribution of journals that had articles published based on abstracts presented at conferences.

Journal name (Brazil)	Number of articles
Journal of Coloproctology	52
Diseases of the Colon & Rectum	8
ABCD: Brazilian Archives of Digestive Surgery	8
Gastroenterology Archives	6
Journal of the Brazilian College of Surgeons	5
Colorectal Disease	3
Brazilian Surgical Record	2
Clinics	2
International Journal of Radiology & Radiation Therapy	2
International Journal of Surgery Case Reports	2
Techniques in Coloproctology	2
Others	29

*Source: Author (2022).*

Over half of the journals (57.7%) are indexed in at least four or five bases, and 2.5% are not indexed in any. The most common database was SCOPUS (81.8%), while Web of Science had the lowest representation (19.8%). We found the impact factor in 89.3% of those published in the global analysis from 2015 to 2019, and the average impact factor according to each evaluation method was 4.23±0.83 for JCR, 0.58±0.13 for SCI, and 46.4±11.86 for H-index; however, the coefficient of variation of this sample was greater than 50% in the three analyses.

The number of abstract authors was similar to that of published works, 6.67±0.48, with a higher level of evidence and better distribution within prospective, retrospective, and experimental studies (Table 2), but none had evidence level 1.

The published works had 7.22±2.70 citations on average, and these were present in only 62% of them. There was no homogeneity, with 92 being the maximum number of citations of a work. In 2019, only 31.3% of the works had citations, compared to 60% in previous years.

We found that publication has a statistically significant relationship (p<0.001) with several factors (Table 1): for oral presentations, 31.3% of unpublished studies and 62% of published ones; for the presence of statistical analysis, 49.1% of the published works versus 31.7% of the unpublished ones; for prospective studies, 11.5% of unpublished and 32.2% of published ones. On the other hand, there were also variables in decline in relation to published works, such as the number of case reports, representing 51.9% of unpublished and 15.7% of published ones.

Table 4 brings the factors associated with publication. In a bivariate analysis, we observed that the following characteristics were statistically significant for publication: category “Awarded”, with prevalence ratio (PR) 4.35 (95% CI 2.11-8.96, p<0.001); being presented in the oral category (PR 3.25, 95% CI 2.32-4.54, p<0.001), presence of statistical analysis (PR 3.43, 95% CI 2.47-4.76, p<0.001); and high level of evidence (PR 3.06, 95% CI 2.16-4.34, p<0.001).

**Table 4:** Bivariate and Multivariate Analysis of the factors associated with the conversion of abstracts presented in papers published in journals.

Variables	Published	Not published	p-value	Bivariate Analysis	p-value	Multivariate analysis
Category				RP		OR
Oral	75	512	<0.001	3.25 (2.32-4.54)	0.301	0.78 (0.48-1.25)
Poster	46	1,123				
University center						
Yes	71	978	0.805	0.96 (0.68-1.36)	0.722	0.92 (0.58-1.46)
No	50	657				
Multicentric						
Yes	17	131	0.019	1.79 (1.10-2.92)	0.013	2.21 (1.18-4.14)
No	103	1504				
Awarded					-	-
Yes	6	10	<0.001	4.35 (2.11-8.96)		
No	84	890				
Presence of statistical analysis						
Yes	50	249	<0.001	3.43 (2.47-4.76)	0.007	2.01 (1.21-3.31)
Variables	Published	Not published	p-value	Bivariate Analysis	p-value	Multivariate analysis
No	52	538				
Published in previous congresses					-	-
Yes	16	43	<0.001	4.38 (2.76-6.97)		
No	105	1,592				
Low	80	1,409				

*Multivariate Logistic Regression Model by Enter Method, using Wald Test. PR: Prevalence Ratio; OR: Odds Ratio.*

Within the multivariate analysis for published studies (Table 4), we observed that multicentric studies and the presence of statistical analysis remained the factors with greater chances of publication, with Odds ratios (OR) 2.21 (95% CI 1.18-4.14) and 2.21 (95% CI 1.21-3.31), respectively, when compared with single-center studies and lack of statistical analysis.

## DISCUSSION

Scientific events are important places for the dissemination and exchange of knowledge, since the presentation of papers at congresses are integral components of research processes and medical education, and offer opportunities for academics, graduate students, and professionals in general to share their results with other researchers and/or event participants. In addition, at these events, undergraduate and graduate students have the chance to practice how to present data with scientific language and formatting both in an oral presentation and on a poster.

Especially at the conference level, abstracts are a cut-off point to decide which papers will or will not be presented at events. In the present study, we analyzed a total of 1,756 abstracts presented at the Brazilian Congress of Coloproctology over a period of five years. The number of papers studied is high when compared with work published on this theme^18^ ^,^ ^19^, with an average of abstracts analyzed of 383, according to a systematic review consisting of 425 articles^8^. The quality of the abstracts submitted in many events also determines the type of presentation to be conducted, the best ones being allocated for an oral presentation, in which the presenter has more time to present and discuss the subject when compared with a presentation poster.

The predominance of the poster category found in our study (66.6%) is similar to the one found by a Turkish study in the area^18^. The oral presentation category is usually reserved for higher quality works, and in our study this factor increased the chance of publication by more than three times (PR 3.25, 95% CI 2.32-4.54, p<0.001). On the other hand, works with a lower level of evidence, the main representative of this group being the “Case Report”, do not require an oral presentation because the poster gathers all the necessary information and because they are presented more quickly, occupying less time and physical space, and requiring a smaller number of evaluators. Previous works, including a British evaluation in coloproctology, also demonstrated this trend, but the literature is not uniform regarding these data^19^ ^-^ ^23^. More complex studies, such as multicenter ones, were also significantly more published in our analysis (OR 2.21, 95% CI 1.18-4.14).

The presentation of papers at conferences is an intermediate step between conducting the research and the final publication, allowing to show preliminary results or to report unusual or unexpected discoveries, which is important for the discussion of innovative scientific topics8. However, these works are presented in a reduced and simplified form, not adequately peer-reviewed, without questioning of methods and results. If, on the one hand, the acceptance of a greater number of works for presentation is without a doubt a stimulus to the medical, academic, and scientific community, on the other, the objective of organizing these events is to raise a greater number of registrations and inflate profits for the organizing society.

In this study, we verified a considerable portion of abstracts of case reports (49.4%) and retrospective assessments (30.4%). In addition, we observed that, from 2017 to 2019, there was a significant decrease in the number of prospective studies, while the number of case reports increased. Case reports only deserve publication when dealing with a rare case or common cases with a rare evolution. This fact results in worse publication perspectives when observing such proportions of this study design, as evidenced in the Brazilian Congress of Coloproctology. In other studies, especially in the areas of Nephrology and Orthopedics^24^ ^,^ ^25^, the opposite has been observed, an improvement in the quality of the level of evidence over the last few years.

The scientific production of works with simpler study designs is more attractive from the curricular point of view, since the retrospective review of medical records, such as the Case Report, has a shorter execution time^26^, requiring less effort and detachment from resources, in addition to being a task easily delegated to less qualified professionals and medical students. This contributes to the greater participation of undergraduates in the scientific event. On the other hand, this type of study has little scientific value and little practical impact. Since congresses are often just a way to boost individual curriculum quality, physicians end up opting for simpler forms of academic production, and this preference has become a growing trend in Brazilian Coloproctology congresses, as observed herein. This data should not be taken as positive, since the production of works without relevance or with a low level of evidence does not add scientific growth to the area, preventing improvements and technical innovations, in addition to uncertainties regarding subsequent publication.

The other characteristics of the abstracts (number of subjects, number of authors) are in line with the bibliometric trends of other surgical conferences^5^ ^,^ ^27^. 

### Abstracts converted to full manuscripts

Although the elaboration and acceptance of an abstract in a medical specialty congress is important for the dissemination of scientific knowledge, the publication of research in complete manuscripts before or after presentation is an essential step for validating data and disseminating findings consistently^7^ ^,^ ^28^.

The importance of scientific research is recognized in Brazil, although not always encouraged, and it has grown significantly in recent years, with an increase in papers published with the names of Brazilian authors in indexed journals^29^ ^,^ ^30^. In addition, surgical areas have a greater volume of publications when compared with other medical areas^31^. However, abstracts presented at different Brazilian medical congresses have been accompanied by a relatively low rate of conversion into publications of full manuscripts in peer-reviewed, indexed journals^4^ ^,^ ^6^. One can list adverse consequences of this inaccessibility of research, which include unnecessary duplication of experiences, delays in the dissemination of advances in patient care strategies, harm to patients, waste of limited resources, and loss of scientific integrity^32^.

The conversion rate from abstracts to full manuscripts is an important indicator of the scientific level of medical conferences^8^ ^,^ ^33^. In Brazil, studies that evaluated these scientific events found variable rates: 16.9% in Oncology^34^, 6.3% in Vascular Surgery^12^, 26.6% in Orthopedics35, 2.6% in General Surgery 4, 39-51.3% in Urology 6,36, and 2.9% in Trauma^37^. Another recent analysis demonstrated that the conversion rates of such abstracts can vary between 11% and 78% in different medical specialties worldwide38. Two systematic reviews have consistently assessed the conversion rate, the first of which (2007) evaluated 29,729 abstracts from different medical areas, with 44.5% of publications^14^. In another analysis also conducted by Cochrane, in 2018, the same author and his collaborators evaluated 307,028 abstracts through 425 reports, demonstrating a drop in the overall conversion rate to 37.3%^8^. In Coloproctology, there was no similar research in Brazil, and the only two published studies refer to the Society of Coloproctology in the United Kingdom, with a conversion rate of 24.3% evaluating a single year of congress (2001), and Turkey, with 22.6% in abstracts evaluated between 2003 and 2011^18^ ^,^ ^19^.

In the present study, the analyzed scientific event is the most important for Coloproctology in Brazil and the second largest in the world in terms of number of participants. We assessed conversion rates in a series of five years, finding only 6.9% of the presented abstracts published. Over the studied years, there was a significant drop in the publication rate, from its highest number in 2015, 11.4%, to 3.8% in 2019. Rejection by journals can be a cause of non-publication. However, studies suggest that most unpublished works have not even been submitted to journals^7^ ^,^ ^8^. More rigorous assessments in the approval of these presented papers or even proposing the submission of complete papers and not just their abstracts could filter out those of lower methodological quality or scientific relevance, predisposing to higher publication rates.

As in Scherer’s meta-analysis, we analyzed a period of more than 24 months between presentation and subsequent publication, and we found that 32.2% of publications occurred before presentation at the congress^8^ ^,^ ^14^. Another 56.2% occurred up to 24 months after the event. Only 11.6% of publications occurred in a period equal to or greater than 25 months from the congress. Such findings are compatible with results found in the literature^8^ ^,^ ^18^.

In our study, 35.5% of the publications were in international journals, the SBCP journal itself receiving 42.9% of the publications in the period. Its publication fee is currently subsidized by the society, making it an attractive factor in choosing the journal. Previous research has shown that the cost of publication is a barrier to not going ahead with research^39^ ^-^ ^42^. Previous investigations have also highlighted that abstracts presented at events organized by scientific societies have preferably been submitted for publication in their official journals^7^ ^,^ ^18^ ^,^ ^19^.

We also investigated abstract-independent factors that may predict publication in full. Such an analysis has not been previously conducted in the literature related to congresses in the area. In our bivariate analysis, award-winning abstracts were 4.35 times more likely to be published (95% CI 2.11-8.96). A similar analysis showed that awarded studies had a conversion rate 66.6% higher than the global average for that event^43^.

The presence of statistical analysis occurred in only 33.6% of the studies, although the appropriate inclusion of these statistical tests is considered an important quality criterion for abstracts^11^. Among the works converted into publication, the presence of statistical analysis occurred in 74.3%, a determining factor for the success of conversion in the present study (PR 3.43, 95% CI 2.47-4.76), in bivariate and multivariate analysis, as well as exhibited in congresses of different societies^10^ ^,^ ^11^ ^,^ ^44^. This shows that correctly analyzed data are essential to test the original research hypothesis^45^ ^,^ ^46^.

The reasons for non-publication have already been studied, but they are not completely clear and are probably multifactorial^47^ ^,^ ^48^. A systematic review describes lack of resources, publication not being an author’s goal, low priority, incomplete study, and problems with co-authors as major factors. However, lack of time was the most frequently reported and most important reason for not publishing abstracts as full manuscripts^42^ ^,^ ^47^. We did not interview the abstract’s authors, so we have no information regarding the reasons for the low conversion rate. 

### Limitations

It is possible that the search strategy did not accurately identify all publications, although the selection, inclusion, and analysis methods were based on similar investigations^1^ ^,^ ^5^ ^,^ ^7^ ^,^ ^9^ ^,^ ^14^ ^,^ ^49^ ^,^ ^50^.

Another limitation is the evaluation of a single niche in a specific time interval, which is the specialty of Coloproctology, and therefore, any generalization of the findings must be limited to temporal and regional biases^15^. Although quantitative data are presented, we performed no additional interpretive analysis (validity, consistency, and/or quality). Consequently, new research should emerge through methodological modifications, such as the inclusion of different potential predictive factors of a higher conversion rate and assessment of the quality of meeting abstracts.

Moreover, data collection lasted just over two years after the last congress evaluated in 2019, which is perhaps a short period to capture manuscript publications. It is possible that some abstracts will eventually be published later and would appear as such with a longer follow-up. Nonetheless, the vast majority (88.4%) of manuscripts were published within 24 months of submission, in line with literature data^7^ ^,^ ^8^ ^,^ ^14^ ^,^ ^18^ ^,^ ^19^. Therefore, it is unlikely that we significantly underestimated the publication rate.

Another caveat stems from the evaluated of the proportional Brazilian contribution in the form of conference abstracts. The conversion rate of abstracts into publications is not the only instrument for measuring the scientific quality of a congress, as it has several other activities for professional updating and dissemination of scientific knowledge. It is possible that superior-quality Brazilian studies were published as complete manuscripts over the studied years, without presentation in the Coloproctology congresses. The results probably underestimate the general Brazilian Coloproctology publication rate.

Other studies like this one, with an auditing and monitoring nature of research practices, should be encouraged in favor of improvements for the medical society of the specialty in question, enabling it to expand its visibility in an international scientific scope and achieve better quality in evidence-based medicine offered to its patients in its national congresses.

## CONCLUSION

Although the SBCP is among the largest in the world, the data presented demonstrate a low scientific productivity of the specialty in its congress, since the research conducted is mostly unpublished, with an average of only 6.9% of the works converted to full published manuscripts in the analyzed years. The predictors of abstract’s publication were related to higher quality and complexity of the works: multicenter studies, studies with statistical analysis, studies with a higher level of evidence, and studies awarded by the congress.

We conclude from the unpublished data presented in this study that efforts are needed to improve the performance of scientific publications of Brazilian Coloproctology. Government, departmental, and SBCP support for both preceptors, residents, and undergraduates, including dedicated research time and research infrastructure, are urgently needed to address such deficiencies.
